# Practical Recommendations for the Selection of Patients for Individualized Splice-Switching ASO-Based Treatments

**DOI:** 10.1155/2024/9920230

**Published:** 2024-04-18

**Authors:** Bianca Zardetto, Marlen C. Lauffer, Willeke van Roon-Mom, Annemieke Aartsma-Rus, on behalf of the N = 1 Collaborative

**Affiliations:** ^1^Dutch Center for RNA Therapeutics, Department of Human Genetics, Leiden University Medical Center, Leiden, Netherlands; ^2^7 Carole Place, Somerville, MA 02143, USA

## Abstract

Although around 6% of the world's population is affected by rare diseases, only a small number of disease-modifying therapies are available. In recent years, antisense oligonucleotides (ASOs) have emerged as one option for the development of therapeutics for orphan diseases. In particular, ASOs can be utilized for individualized genetic treatments, addressing patients with a known disease-causing genetic variant, who would otherwise not be able to receive therapy. Careful prioritization of genetic variants amenable to an ASO approach is crucial to increase chances for successful treatments and reduce costs and time for drug development. At present, there is no consensus on how to systematically approach this selection procedure. Here, we present practical guidelines to evaluate disease-causing variants and standardize the process of selecting *n*-of-1 cases. We focus on variants leading to a loss of function in monogenic disorders and consider which splice-switching ASO-mediated treatments are applicable in each case. To ease the understanding and application of our guidelines, we created a hypothetical transcript covering different pathogenic variants and explained their evaluation in detail. We support our recommendations with real-life examples and add further considerations to be applied to specific cases to provide a comprehensive framework for selecting eligible variants.

## 1. Introduction

Rare diseases are defined as conditions affecting less than 1 in 2,000 individuals within Europe. However, with an estimated 7,000 different rare diseases [1], approximately 470 million people are affected worldwide [2]. Although a genetic origin has been identified for more than 70% of these diseases [3], less than 6% currently have approved disease-modifying treatments [4, 5]. Affected individuals are often refractory to drugs commonly used for the management of clinical manifestations [6], illustrating the urgency of developing targeted treatments.

Recent progress in RNA-based therapies has shown that antisense oligonucleotide- (ASO-) mediated treatments offer the potential to partially fill this therapeutic gap [7]. For ultrarare disorders, ASOs represent an opportunity to design a disease-modifying drug in a patient-specific fashion. As of now, 19 oligonucleotide-based therapies have been approved by either the European Medicines Agency (EMA), the Food and Drug Administration (FDA), or the Japanese Ministry of Health, Labour and Welfare [8], 10 of which are ASOs. The case of an *n*-of-1 ASO treatment for a single patient with neuronal ceroid lipofuscinosis (milasen) has paved the way for the development of individualized treatments targeting private variants [9, 10].

With the increasing use of whole-genome sequencing (WGS) and other advanced diagnostic methods, more of these unique pathogenic variants are being identified. However, not all are amenable to treatments using ASO-based approaches. Careful consideration of each case is required to ensure that resources are focused on promising targets. There is often only a narrow window of opportunity for treatment development and initiation as patients will progress further during the development process, increasing the urgency for timely drug delivery and being specific in whom to select for targeted treatment.

Here, we provide practical recommendations for the assessment of pathogenic variants for individualized genetic treatments and explain which variants are eligible for customized approaches. We will focus on one particular type of ASOs, called splice-switching ASOs (ssASOs).

## 2. Splice-Switching ASOs

ASOs are short, single-stranded pieces of chemically modified nucleic acids. They bind to their target, the (pre-)mRNA, through canonical Watson-Crick base pairing and can modify protein expression via different mechanisms [11]. ASOs can be developed to treat large groups of patients but can also be custom-made for individual cases. There are two main mechanisms of action used for modifying the target transcript, namely, steric blocking (splice-switching) and transcript reduction via RNase H-mediated RNA degradation [12]. The latter approach is used to downregulate transcript levels and is mainly employed in diseases associated with toxic gain-of-function (GoF) mechanisms. Another option to downregulate transcripts in diseases associated with toxic GoF variants is siRNAs (small interfering RNAs) [13]. Gapmer ASOs and siRNAs are not specific to a single case. Hence, for these guidelines, we will only consider splice-switching ASOs that can be used for individual variants.

ssASOs regulate gene expression by impeding the interaction of splicing factors with regulatory sequences in the pre-mRNA [7]. Such regulatory elements are, for example, the canonical splice sites at the exon-intron/intron-exon boundaries and the branch point, as well as exonic/intronic splice enhancers and silencers [14, 15].

Through restoration of the open reading frame of an RNA transcript, ssASOs are able to fully or partially rescue protein function. This can be achieved through different mechanisms, such as skipping exons (including cryptic exons) and exon inclusion. ASOs are also able to influence protein levels by targeting the untranslated regions (UTRs) [16, 17]. Here, the ASOs are not modifying splicing, but by sterically blocking regulatory elements, they can influence the stability of a transcript. ssASOs have been used to treat groups of patients, like in spinal muscular atrophy, where nusinersen is used for exon inclusion, as well as for single patients with private disease-causing variants as seen with Mila, a patient with Batten's disease [9, 18].

## 3. Practical Guidelines

### 3.1. Introduction to Practical Guidelines

The following guidelines apply to monogenic diseases where the disease-causing variants have been identified and classified as (likely) pathogenic according to the ACMG criteria [19]. For splice-altering variants, the splice-disrupting effect must have been confirmed functionally, for example, via RT-qPCR or RNA-seq analysis. The guidelines focus on loss-of-function (LoF) variants, where a restoration of the reading frame and production of a (partially) functional protein is the aim. For GoF variants and variants causing a dominant-negative effect, different considerations apply. These are briefly discussed later. The guidelines are restricted to single nucleotide variants, small indels, and single-exon deletions. Deletions and insertions of larger parts of genes, copy number variants, and repeat expansions cannot be evaluated using the outlined recommendations. We further only consider variants that can be targeted with a single ASO. General considerations regarding which type of disease and pathomechanism are treatable with which ASO approach have been discussed by us elsewhere [20].

**Figure 1:**
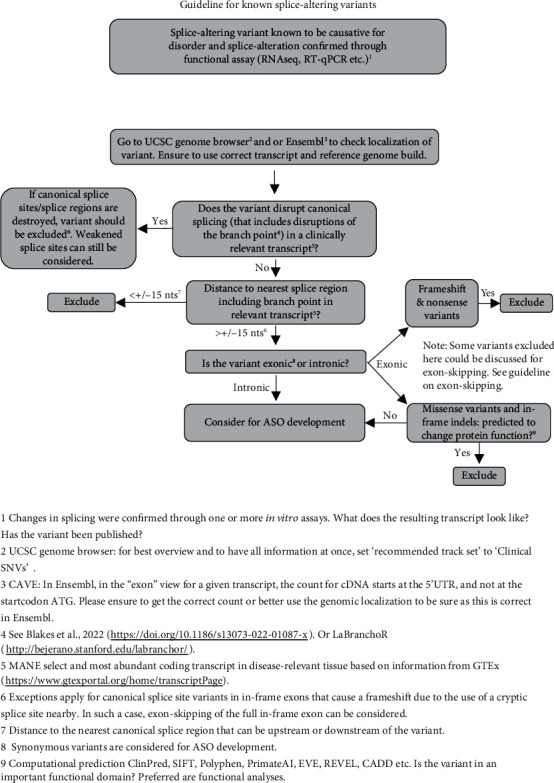
Decision tree for variant evaluation of variants known to disrupt splicing by creating or activating cryptic splice sites. The aim of this decision tree is to identify cryptic splice variants that can be corrected with a ssASO by blocking the cryptic splice site to restore the open reading frame.

**Figure 2:**
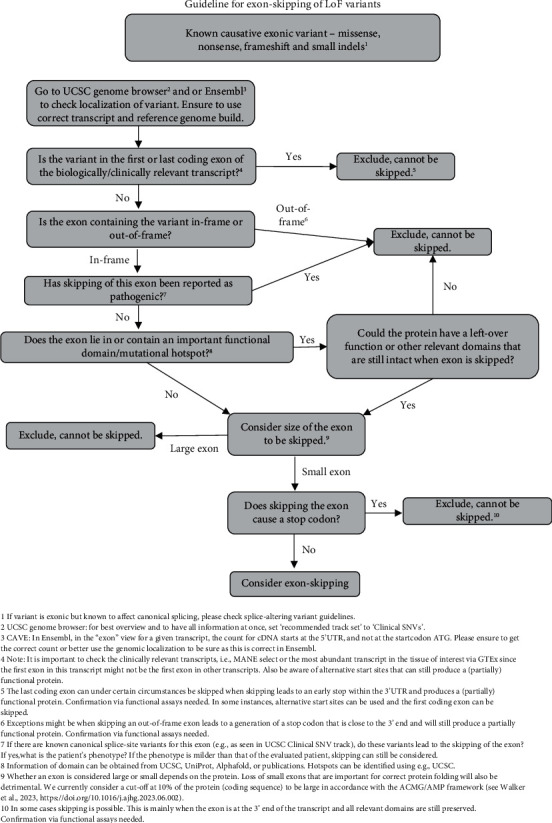
Decision tree for variant evaluation of exonic LoF variants not disrupting splicing. The aim of this decision tree is to identify exonic LoF variants that can be targeted with a ssASO via exon skipping.

Our recommendations for variant selection are based on guidelines established by the Dutch Center for RNA Therapeutics in the form of decision trees (Figures 1 and 2). These are meant to aid in recognizing those unique pathogenic variants that can be targeted by ssASOs. Two different decision trees have been designed. The one depicted in Figure 1 is aimed at variants known to disrupt splicing by creating or activating cryptic splice sites. By blocking the variant with a ssASO, canonical splicing can be restored, and the full protein product produced. The decision tree shown in Figure 2 is aimed at exonic LoF variants that do not disrupt splicing. For these variants, exon skipping can be considered. Here, a ssASO is used to mask an exon from the splice apparatus to produce a restored reading frame, an internally truncated transcript, and, eventually, an internally truncated (partially) functional protein. To decide whether an exon can be skipped, a multitude of parameters need to be collected (Figure 2).

### 3.2. LoF Variant Overview

**Figure 3:**
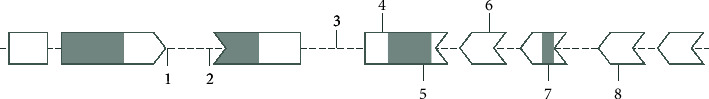
Hypothetical transcript containing different loss-of-function variants. The shape of each exon depicts the reading frame. Grey areas within the exons show important functional domains. Variants are as follows: (1) canonical splice site variant; (2) branch point variant; (3) intronic cryptic splice site variant; (4) exonic cryptic splice site variant; (5) variant disrupting a splice-regulatory element; (6) variant leading to an early truncation, i.e., nonsense variants and frameshift variants; and (7) and (8) missense variants. The shape of the exons represents the reading frames, and grey marked areas are protein domains.

We will use a hypothetical transcript (Figure 3) containing 8 different variant types, all leading to loss of protein function, that can be evaluated using the decision trees. The following section will focus on explaining the assessment of each variant individually, visualizing the variant and potential treatment effects on transcript and protein level. We provide real-life examples for the different variant types.

#### 3.2.1. Canonical Splice Site Variants and Splice Region Variants

Genetic alterations that occur at the canonical splice sites, i.e., the boundary of the exon-intron/intron-exon junctions (+1,+2 splice donor dinucleotide and -1,-2 splice acceptor dinucleotide), are identified as canonical splice site variants. Such variants can destroy or severely weaken the canonical splice sites. Variants that destroy canonical splicing can also occur upstream or downstream of the canonical splice sites. This is especially the case for the splice site regions, i.e., -3 to +6 for the splice donor region and -20 to +1 for the splice acceptor region, including the polypyrimidine tract [21].

**Figure 4:**
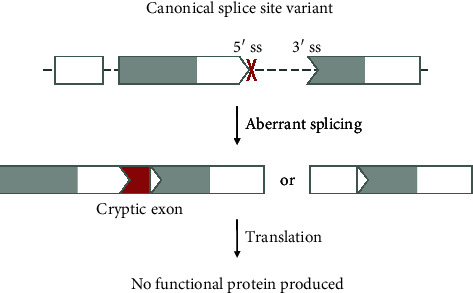
Variant 1—canonical splice site variant. Pathogenic variants that destroy or severely weaken the 5′ donor or 3′ acceptor sites often result in intron retention (cryptic exon) or (partial) exon skipping. This can lead to disruption of the reading frame of the transcript and reduced protein production. As the splicing machinery cannot recognize destroyed splice sites, normal splicing cannot be restored with an ASO. The shape of the exon indicates the reading frame, and grey marked areas are protein domains. ss: splice site.

ssASOs cannot be used to restore normal splicing when the canonical splice site is disrupted. In such situations, the splice site cannot be recognized by the splicing machinery anymore, leading to a disruption of constitutive splicing in itself (Figure 4). Splice site variants can cause (partial) exon skipping, intron retention, or altered splicing of multiple exons [22]. Variants causing part of an exon to be skipped could be considered partial exon skipping events. However, it is also possible for the canonical splice site to get weakened and be recognized by the splicing apparatus less frequently, leading to the inclusion of that exon only in a small percentage of the transcripts. To distinguish between these two forms of “partial exon skipping,” we refer to the event where part of an exon is excluded in all transcripts as cryptic splicing and the event where the whole exon is excluded at a certain percentage of the transcripts as partial exon skipping.

The exact effect of a canonical splice site variant has to be determined with a functional analysis, that is for example RT-qPCR or RNAseq.

There are a plethora of examples of pathogenic canonical splice site variants, one of whom is the NM_001371596.2(MFSD8):c.754+2T>A variant identified in patients with childhood dementia. Although the disease presentation is highly eligible for an ASO-based therapy, the variant is not. This variant was shown to lead to an alternative splicing pattern, with the normal transcript being almost completely lost [23]. While ssASOs may be used to block the use of alternative splicing patterns, this will most likely not restore normal splicing as the splice site is not functional.

Notably, if a canonical splice site variant leads to the skipping of a complete out-of-frame exon without further disruptions of the transcript, the variant can be seen as causing a single-exon deletion leading to a frameshift. Here, an ASO-based strategy aiming at skipping adjacent exons to correct the reading frame could be considered.

It should be stressed that pathogenicity needs to be confirmed also for variants occurring at the canonical splice site or within the splice region, ideally with a functional assay. Recent studies have shown that not all splice site variants are disease-causing, and some do not affect splicing at all [21, 24].

Additionally, the effect of the variant should be carefully evaluated as not all splice region variants cause skipping of the exon but might have unusual effects that could be approached with a ssASO. An example is the NM_025233.7(COASY):c.1486-3C>G variant within the splice acceptor region of exon 8. Counterintuitively, the variant causes skipping of exon 7 and partial retention of intron 7 due to activating a cryptic splice acceptor site that lies 53 nucleotides upstream of exon 8 [25]. Blocking this cryptic splice acceptor site with a ssASO would be one therapeutic option.

In summary, splice site and splice region variants that destroy or severely weaken the splice site and lead to exon skipping are no suitable targets for ssASOs.

#### 3.2.2. Branch Point Variants

**Figure 5:**
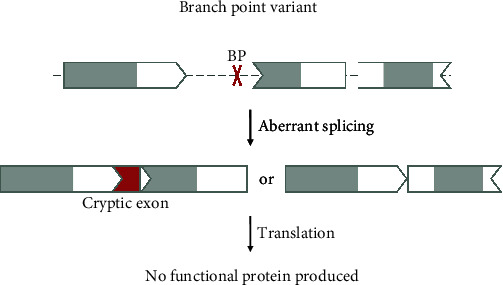
Variant 2—branch point variant. Pathogenic variants disrupting the branch point can affect splicing and lead to the inclusion of a cryptic exon by activating a cryptic splice site or skipping of the canonical exon. Ultimately, this causes a loss of function of the protein. Since the canonical sequence required for correct splicing is destroyed, these variants cannot be rescued via ssASOs. The shape of the exon indicates the reading frame, and grey marked areas are protein domains.

The branch point is a cis-acting intronic sequence that lies about 18 to 40 nucleotides upstream of the 3′ end of an intron and is crucial during the splicing reaction [26]. Variants at or around the branch point can also influence canonical splicing and cause disease (Figure 5) [27].

Here, the same considerations as for canonical splice site variants apply. If variants at or around the branch point destroy or severly disrupt the branch point, these variants cannot be targeted with a ssASO.

It is difficult to predict whether a variant is destroying or disrupting a branch point, and the altered splicing pattern of a mutated transcript is best analyzed using functional assays. Tools like LaBranchoR can help determine splicing branch points [28]. At this point, variants lying upstream of the 3′ end of an intron within the region where branch points can usually be found should be considered a potential branch point disrupting variant. It should also be noted that if a variant within this region is causing cryptic splicing, targeting the pathogenic variant with a ssASO can disrupt the branch point and interfere with canonical splicing.

An example of a variant affecting the branch point is NM_025152.3(NUBPL):c.815-27T>C. This variant lies in intron 9 of the *NUBPL* gene and leads to different effects. It causes skipping of exon 10 altogether as well as the activation of a cryptic splice site, eventually leading to nonsense-mediated decay of that transcript [29]. It is thus unlikely amenable to a ssASO strategy.

#### 3.2.3. Intronic Cryptic Splice Site Variants

**Figure 6:**
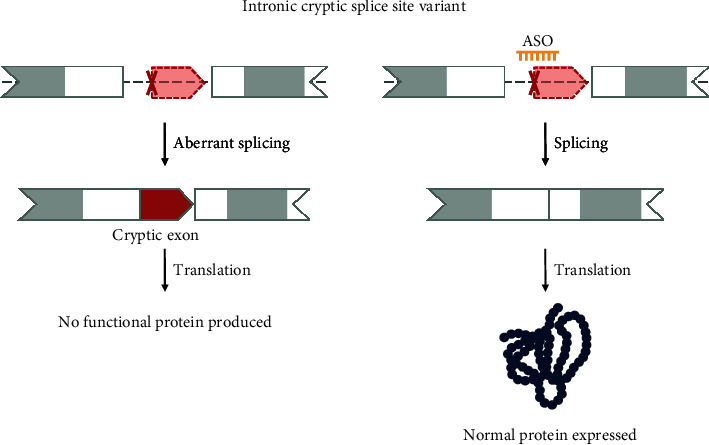
Variant 3—intronic cryptic splice site variant. Deep intronic variants can create or activate a cryptic splice site, leading to the integration of part of the intron (cryptic exon) into the mRNA and an early truncation. By targeting the cryptic splice site with ssASOs, it is possible to mask the cryptic splice site and restore the full-length native transcript and, thus, expression of the canonical protein. The shape of the exon indicates the reading frame, and grey marked areas are protein domains.

Intronic variants that create or activate cryptic splice sites are responsible for the incorporation of parts of the intron into the mRNA, called a cryptic exon (Figure 6). This will often lead to the creation of an early stop and thus aberrant or no protein production. By targeting the intronic cryptic splice site with a ssASO and masking the cryptic exon from the splicing apparatus, the original transcript can be restored, and the physiological protein can be produced [30]. For an intronic cryptic splice variant to be eligible for ssASO treatment, it should not affect canonical splicing and be sufficiently removed from the canonical splice regions and the branch point (Figure 1). It should also be possible to design a ssASO that targets the pathogenic variant without it disrupting canonical splicing. We thus advise that the intronic variant is at least 5 nucleotides (hard cut-off) or, better, 15 nucleotides (recommended cut-off) removed from the nearest splice site/branch point to ensure that canonical splicing can be restored. Ideally, variants within this category are deep intronic, with a defined distance of >100 nucleotides from the nearest exon junction [31].

Multiple ssASOs have been developed for deep intronic cryptic splice sites, one example is milasen [9].

Another example of a suitable deep intronic variant to be targeted by a ssASO is NM_016589.4(TIMMDC1):c.597-1340A>G. This variant has been associated with a severe neurodegenerative disorder starting from the early postnatal period. Indeed, an ASO targeting the variant has already been shown to correct canonical splicing *in vitro* [32].

By targeting deep intronic cryptic splicing variants with ssASOs canonical splicing can be restored, and normal protein production can be expected, This reduces the need for excessive functional studies on protein function after ASO treatment. Unfortunately, the deep intronic variants are currently often missed by routine genetic diagnostic screenings. Diagnostic pipelines are primarily focused on sequencing the exome, and where genomic data is available, we are lacking tools to sufficiently predict intronic splice-disrupting variants.

#### 3.2.4. Exonic Cryptic Splice Site Variants

**Figure 7:**
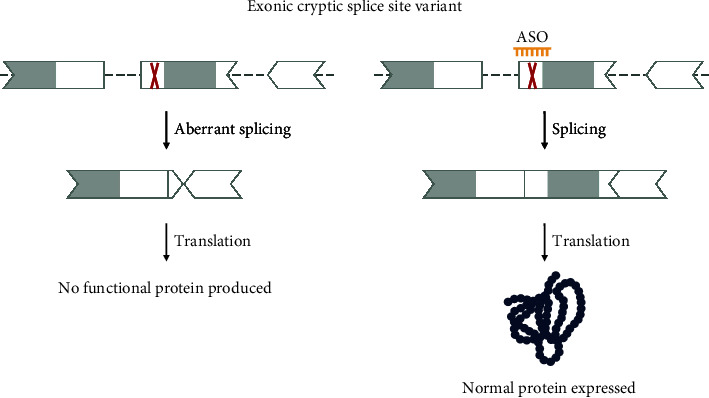
Variant 4—exonic cryptic splice site variant. Exonic variants can create or activate cryptic splice sites, leading to the exclusion of part of the exon and no functional protein production. The variant site can be targeted with a ssASO to hide the cryptic site, thus restoring splicing and protein production. The shape of the exon indicates the reading frame, and grey marked areas are protein domains.

Exonic variants that create or activate a cryptic exonic splice site change the splicing pattern by incorporating only parts of the exon into the mRNA transcript, mainly causing a disruption of the reading frame and an early stop (Figure 7). Missense, nonsense, and synonymous variants as well as small indels can lead to exonic cryptic splicing, however, the ideal candidates to be considered for these cases are synonymous variants that solely affect splicing.

Compared to the (deep) intronic cryptic splice site variants, exonic cryptic splicing variants need to be assessed with more caution. The effect of the cryptic splice variant should be determined using a functional assay. Similarly to what we discussed for intronic cryptic splice site variants, the exonic variant should be sufficiently removed from the canonical splice sites to not impact canonical splicing and for a ssASO to bind without disrupting the splicing machinery. Also for these variants, we recommend keeping a distance of 15 nucleotides from the nearest canonical splice site. Nonsynonymous variants should be handled with caution as the amino acid change in itself, and not only the effect on splicing, can be disease causing. In such instances, correction of the splicing pattern will not be therapeutic. Note, should the variant be located in an exon that qualifies for exon skipping (see Section 3.2.6), skipping of the complete exon can be considered.

Multiple exonic cryptic splice sites have been identified over the years, and correction of the aberrant splicing pattern using ssASOs has successfully been demonstrated. One such variant is the NM_000051.4(ATM):c.7865C>T (p.Ala2622Val) variant identified in patients with ataxia telangiectasia, a disease very much suited for ASO treatment [33]. A ssASO named atipeksen has been developed to target this variant in patients [10].

It should be noted that designing ASOs to target an exonic cryptic splice site can also result in skipping of the exon altogether and must be carefully evaluated during the preclinical assessment.

#### 3.2.5. Variants Disrupting Splice-Regulatory Elements

**Figure 8:**
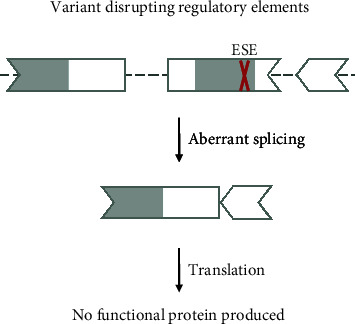
Variant 5—variant disrupting a splice-regulatory element. Exonic variants can disrupt regulatory sequences that promote the canonical splicing process and lead to exon exclusion and disruption of the transcript. Therefore, an exon skipping approach is not applicable. Targeting the variant directly would also not rescue splicing, as the canonical regulatory sequence cannot perform its function. The shape of the exon indicates the reading frame, and grey marked areas are protein domains. ESE: exonic splice enhancer.

Variants can also be disease-causing via the disruption of splice-regulatory elements such as exonic splice enhancers (Figure 8). This will ultimately alter the canonical splicing mechanism and can lead to skipping of the exon during splicing. The variants disrupting regulatory elements can be exonic as well as intronic. Variants that disrupt canonical splicing through this mechanism are unlikely amenable to splice-switching treatment approaches and not amenable to exon skipping. Only in exceptional cases can skipping of an exon caused by the disruption of a splice enhancer be counteracted by targeting splice silencers with a ssASO. This is called exon inclusion (see Section 3.5).

An example of a synonymous variant causing exon skipping via disruption of a regulatory element is the NM_152778.3(MFSD8):c.750A>G (p.Glu250=) variant identified in patients with neuronal ceroid lipofuscinosis [34]. The identified variant was shown to be located within an exonic splice enhancer and leads to skipping of exon 8 or exons 7 and 8 of the transcript. Correction of splicing in this case with a ssASO has not yet been tested.

#### 3.2.6. Nonsense and Frameshift Variants

Variants that create a stop codon and small indels that lead to a frameshift are interesting candidates for an ASO treatment using exon skipping. Here, the variant and the exon wherein the variant is located have to fulfill specific criteria (Figure 2). The variant should lead to a loss of function of the protein either through nonsense-mediated decay of the transcript or the production of an unstable, truncated, and nonfunctional protein (Figure 8). Exon skipping can be considered if the variant is not in the first and/or last coding exon, and the exon is in-frame, meaning that the length of the exon in nucleotides is divisible by 3. The exon should also not contain any relevant functional domains. This might not always be known since not all genes/proteins associated with rare diseases are well-studied. Which domains are crucial for the protein to function or necessary for partial functionality is different for every protein.

Additionally, proteins containing repeat domains might be tolerant to the loss of one or multiple of the repeat domains. We recommend contacting an expert on the protein for a second opinion. Extensive functional testing will also be required to determine restoration of protein function and rule out whether skipping the exon does not cause other damaging effects, for example, a gain of function. Which functional analysis is required to provide sufficient evidence of a positive treatment effect of exon skipping is again highly dependent on the protein in question and its overall function. Such analyses can include assays on protein localisation, enzymatic function, or downstream analyses like restoration of electrophysiological patterns in neuronal networks. We recommend talking to experts within the field to establish suitable functional assays.

Further, the size of the exon should be considered. While there are no general rules as to what exon length is considered too large for skipping, some guidance is available. The ACMG/AMP framework considers an in-frame deletion of >10% of the protein a strong criterion for predicted loss of function [35]. However, some proteins are partially functional when missing 30% or more of the coding sequence [36, 37]. Thus, we would like to emphasize that defining small and large exons is highly dependent on the gene/protein of interest, and separate recommendations might be necessary for each gene. This is in line with the recent extension of the ACMG/AMP framework [21]. Assessing exon skipping of in-frame exons also depends on which parts of the protein are being removed and how this can affect folding and, ultimately, function. We expect that new algorithms and prediction tools will help with these steps in the future. Moreover, it is also important to consider the consequences of skipping an exon with respect to the transcript sequence. Since exon boundaries are not always in line with the codons, the first or second nucleotide of a codon can be located at the exon boundary. Removing an in-frame exon could then also lead to the formation of a stop codon or an amino acid change on the new exon-exon junction.

**Figure 9:**
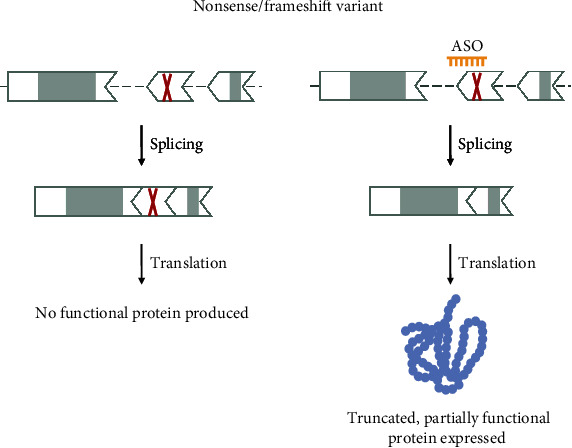
Variant 6—variant leading to an early truncation (nonsense and frameshift variants). Nonsense variants and small indels that are not a multiple of 3 can lead to the generation of an early stop signal in the mRNA transcript and no protein production. The variant-containing exon can be targeted via ssASOs to remove it from the transcript to restore the reading frame. This allows the production of a truncated, partially functional protein. The shape of the exon indicates the reading frame, and grey marked areas are protein domains.

Additional evidence of whether an exon can safely be skipped can be gained from population data. If the exon is known to be deleted in healthy individuals, skipping the exon might be safe. Concurrently, if skipping or deletion of the exon has been identified as a pathogenic, disease-causing variant, it is unlikely that skipping the exon will be therapeutic. Especially for the latter condition, we recommend checking whether there are any reported pathogenic canonical splice site variants for the exon in question and what phenotype the individual carrying such variants has. Ultimately, skipping an in-frame exon that contains a nonsense/frameshift variant will lead to a shortened mRNA and an internally truncated protein product, but the reading frame will be restored (Figure 9).

For example, the NM_014844.5(TECPR2):c.1319del (p.Leu440fs) variant leads to a frameshift and a premature stop. Pathogenic variants in the *TECPR2* gene are associated with an ultrarare neurodegenerative disorder and, thus, interesting candidates for ssASO treatments. An ASO targeting the exon-containing variant was shown to lead to skipping of exon 8 and restored protein production [38].

Of note, if a patient with an exon deletion/skipped exon has a very mild phenotype, milder than that of a patient with a nonsense/frameshift variant, skipping the exon can still be considered as it might mitigate the disease phenotype of the patient. Gene-disease associations for which milder phenotypes in patients with in-frame deletions were observed include *DMD*, *COL2A1*, and *DYSF* [39–41].

#### 3.2.7. Missense Variants and Small In-Frame Indels

Pathogenic missense variants and small in-frame indels are the most difficult variants to evaluate for ssASO treatments. When a missense variant is causing a LoF that leads to disease, it is likely because the original amino acid had a specific function or the missense variant lies within an important (functional) region of the protein. This can, for example, be a functional domain or a linker connecting two domains. That means if a missense variant causing a single amino acid change within an exon renders the protein nonfunctional, it is likely that skipping that exon will also cause a loss of function and render the protein nonfunctional. Similar considerations apply to small in-frame indels.

It could also be that the missense variant is creating or activating an exonic cryptic splice site (see 3.2.4) or disrupting regulatory elements (see 3.2.5), for which functional evidence is necessary.

**Figure 10:**
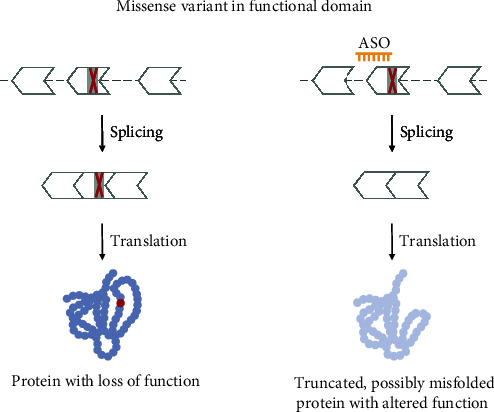
Variant 7—missense variant in an in-frame exon within a functional domain. Missense variants located in regions coding for important domains can disrupt protein function. These variants are not eligible for an exon skipping approach. While removing the variant-containing exon would not disrupt the reading frame of the transcript, it would likely lead to the production of an internally truncated protein with an altered function. The shape of the exon indicates the reading frame, and grey marked areas are protein domains.

For evaluating if an exon containing a missense variant is amenable for exon skipping, the conditions listed under 3.2.6 apply. That is, the exon should be in-frame, not be the first or last coding exon, small enough to be skipped, and not contain an important functional domain. These conditions already lead to the exclusion of variant 7 on the hypothetical transcript (Figure 10). This variant is within a small, in-frame exon but lies within a functional domain. It is thus not eligible for exon skipping.

**Figure 11:**
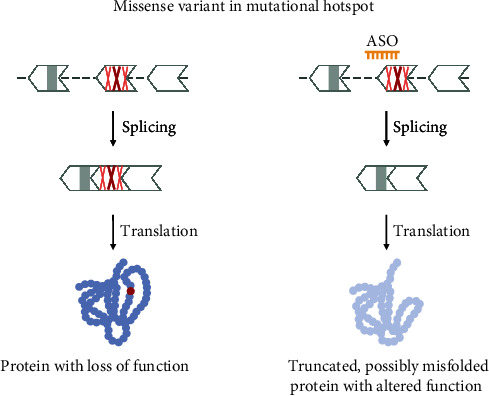
Variant 8—missense variant in mutational hotspot. Missense variants in exons of yet unknown function can lead to the expression of an altered protein. Especially if many missense variants are present in the surrounding region, exon skipping would remove an important function and not produce a therapeutic effect. The shape of the exon indicates the reading frame, and grey marked areas are protein domains.

For variant 8, additional considerations apply. As described for the truncating variants, evaluating whether the exon is skipped naturally in the healthy population (advantageous for exon skipping considerations) or has been identified as skipped in patients (exon not eligible for skipping) will further narrow down potential ssASO candidates. When the variant is within an in-frame exon and no functional domain is known, analyzing the mutational landscape surrounding the variant/within the exon will provide more evidence as to the function of an exon. If most of the neighbouring pathogenic variants are missense, the exon likely encodes a domain/region within the protein with an important function. Often, for the variant itself or the surrounding variants, some analysis of protein function has been performed. If the variant or the surrounding missense variants are known to cause a LoF effect of the protein, then skipping the whole exon will most likely lead to the same, if not a more severe, effect and hence, not be therapeutical. Variant 8 in the hypothetical transcript is within a small, in-frame exon but lies in the middle of a mutational hotspot (Figure 11) and can thus not be considered for an exon skipping therapy.

One example of such a variant is NM_006245.4(PPP2R5D):c.592G>A (p.Glu198Lys). It is located in an in-frame exon with other missense variants in close proximity [42]. The variant has further been tested functionally and shown to have a LoF effect *in vitro* [43]. Skipping the exon containing the variant is, therefore, not a valid therapeutic option.

### 3.3. Toxic Gain-of-Function and Dominant-Negative Variants

Exon skipping can also be used to target toxic GoF or dominant-negative (DN) variants [44]. Following the above-mentioned rules, the exon carrying the variant can be skipped, thereby removing the toxic function and producing a truncated protein. Ultimately, *in vitro* functional assays will have to be performed to validate the residual function of the protein following exon skipping. However, other ASO-based approaches are also applicable to GoF and DN variants. We are discussing them elsewhere [20].

### 3.4. Allele-Specificity

The ssASOs designed for the discussed approaches are usually not allele-specific, meaning both alleles will be targeted. In case of cryptic splice variants, allele-specificity is not needed. While the second, not mutated, allele will be targeted, the ssASO will not have any effect. In case of exonic, heterozygous variants, allele-selective ASOs should be considered if the ASO can negatively impact the splicing of the healthy (wild type) allele. This can, for example, be the case for skipping small, in-frame exons that lead to a truncated, partially functional protein. Here, it might be preferable to keep the wild-type allele intact and have the exon skipped solely on the mutant allele.

### 3.5. Exon Inclusion

While ssASOs are currently mainly used for skipping canonical or cryptic exons, they can also be employed for exon inclusion. Only limited examples of successful exon inclusion are published, such as the inclusion of exon 7 into the *SMN2* transcript (NM_017411.4) for the treatment of patients with spinal muscular atrophy, inclusion of exon 2 in *GAA* (NM_000152.5) for a recurring variant causing Pompe disease, or inclusion of exon 8 for a common *SLC26A4* (NM_000441.2) variant causing sensorineural hearing loss [45–47].

Developing compounds for exon inclusion is far more challenging than developing an ASO for (cryptic) exon skipping [10]. To successfully achieve exon inclusion, cis-acting splice-regulatory elements within or close to the exon have to be identified. That means if the disruption of an exonic splice enhancer leads to exon skipping, exon inclusion can only be promoted when there is an exonic splice silencer or intronic splice silencer working in tandem. Such elements are not always present.

## 4. Patient Selection

Identifying variants most amenable to an individualized treatment is only one part of selecting suitable candidates for ASO-based approaches. The selection process also includes the evaluation of the disease and the patient's phenotype.

While the variant selection is independent of the disease and the individual's clinical presentation, we are currently limited to the type of disease and tissue we can target with ASOs.

To decide which patients qualify for treatment, the following conditions need to be fulfilled. The disease should be life-threatening or severely debilitating, thus warranting an accelerated drug development process. The disorder should be monogenic and predominantly affect the brain, spinal cord, or eye. These tissues can easily be reached through local treatment (i.e., intrathecal, intraventricular, intravitreal, and subretinal injections) to achieve high local exposure while maintaining a low dose and treatment frequency [48].

Further, to allow for the development of an individualized ASO, the individual should still be in a treatable stage two years after enrolment, i.e., the time it will likely take to develop the ASO. If it is to be expected that a patient will deteriorate massively within two years, the patient is unlikely to benefit from individualized treatment. This also means that at the enrolment stage, not all important functions should be lost, as lost function cannot be restored. Importantly, ASO treatments cannot be used to cure the disease, and only affected individuals for whom it is possible to predefine clinical outcome measures can be selected [49]. Treatments are aimed at improving the quality of life by ameliorating the phenotype or slowing disease progression. For example, neurological presentations that can be modified with ASO therapies include the frequency and duration of seizures, myelination levels, and frequency of psychotic episodes.

## 5. Concluding Remarks

The field of individualized ASO treatments for ultrarare diseases is still in its infancy yet rapidly growing. The first step in developing personalized strategies is meticulous target selection, which is integral to the success of the treatment. At present, with such novel developments, there is no established framework for identifying suitable candidates. We here provided practical recommendations to evaluate, in a case-by-case manner, which variants are amenable for ssASO treatments. These guidelines will have to be re-evaluated on a continuous basis, taking the increasing knowledge we gain from treating individuals into account for future adaptations.

As outlined, variants that create or activate cryptic splice sites, both exonic and intronic, represent ideal candidates for these therapeutic approaches. Unfortunately, their characterization remains challenging. For splice-altering variants, distinguishing pathogenic variants from benign ones is less straightforward compared to variants that disrupt the coding sequence, also due to the lack of understanding of their functional consequences. Furthermore, deep intronic disease-causing variants are still largely underrepresented and often overlooked.

Generally, improved diagnostic methods are needed to achieve both earlier diagnosis and higher sensitivity of identification. The routine use of whole-genome sequencing would certainly expand the number of variants detected, especially in noncoding regions. However, it still does not provide the information required for their interpretation. Additional tools and assays are necessary to assess pathogenicity. Several *in silico* prediction models are available [50], but at the moment, it is not possible to select one that performs consistently for all genes or splice-altering variants [51]. To establish usage in the clinical setting, prediction algorithms will have to reliably predict significance and prioritize promising splice-altering variants for further functional testing. RNA sequencing, in particular, can improve the diagnostic yield by helping to elucidate the consequences of variants of unknown significance or cases with no clear exonic candidate [52]. However, analysis is mainly performed on patient-derived blood cells or fibroblasts, which represents a limiting factor, especially for neurological disorders, as around 30% of disease-relevant genes are not expressed in cultured fibroblasts [53]. Further work is required to implement new, improved tools in the diagnostic pipeline, which will eventually facilitate the prioritization of disease-causing variants and, consequently, increase the identification of targets for personalized ASO therapies.

*n*-of-1 strategies are only now emerging as a feasible treatment option for ultrarare disorders, but we can foresee an increase in demand as the field expands. ASOs are highly versatile, and different treatment modalities can be adapted to rescue protein function. Yet, ASO treatments are not always applicable, and eligibility depends on the variant, disease, stage of progression, or patient compliance. Therefore, it is of utmost importance to carefully determine which ASO approach would bring the greatest benefit and whether it is feasible for the case in question.

In conclusion, defining a suitable candidate for individualized ASO treatments requires careful consideration. We here present a set of guidelines to evaluate LoF variants for their amenability for ssASO treatments.
